# Testosterone Plus Low-Intensity Physical Training in Late Life Improves Functional Performance, Skeletal Muscle Mitochondrial Biogenesis, and Mitochondrial Quality Control in Male Mice

**DOI:** 10.1371/journal.pone.0051180

**Published:** 2012-12-11

**Authors:** Wen Guo, Siu Wong, Michelle Li, Wentao Liang, Marc Liesa, Carlo Serra, Ravi Jasuja, Andrzej Bartke, James L. Kirkland, Orian Shirihai, Shalender Bhasin

**Affiliations:** 1 Department of Medicine, Boston University School of Medicine, Boston, Massachusetts, United States of America; 2 Department of Internal Medicine, Southern Illinois School of Medicine, Springfield, Illinois, United States of America; 3 Robert and Arlene Kogod Center on Aging, Mayo Clinic, Rochester, Minnesota, United States of America; Clermont Université, France

## Abstract

Testosterone supplementation increases muscle mass in older men but has not been shown to consistently improve physical function and activity. It has been hypothesized that physical exercise is required to induce the adaptations necessary for translation of testosterone-induced muscle mass gain into functional improvements. However, the effects of testosterone plus low intensity physical exercise training (T/PT) on functional performance and bioenergetics are unknown. In this pilot study, we tested the hypothesis that combined administration of T/PT would improve functional performance and bioenergetics in male mice late in life more than low-intensity physical training alone. 28-month old male mice were randomized to receive T/PT or vehicle plus physical training (V/PT) for 2 months. Compare to V/PT control, administration of T/PT was associated with improvements in muscle mass, grip strength, spontaneous physical movements, and respiratory activity. These changes were correlated with increased mitochondrial DNA copy number and expression of markers for mitochondrial biogenesis. Mice receiving T/PT also displayed increased expression of key elements for mitochondrial quality control, including markers for mitochondrial fission-and-fusion and mitophagy. Concurrently, mice receiving T/PT also displayed increased expression of markers for reduced tissue oxidative damage and improved muscle quality. Conclusion: Testosterone administered with low-intensity physical training improves grip strength, spontaneous movements, and respiratory activity. These functional improvements were associated with increased muscle mitochondrial biogenesis and improved mitochondrial quality control.

## Introduction

In men, age-related decline in serum testosterone levels has been associated with loss of skeletal muscle mass, strength, and physical performance [1], [2], [3], [4], [5], [6]. Randomized clinical trials are in agreement that testosterone supplementation increases skeletal muscle mass in young as well as older men [2], [3], [4]. Indeed, testosterone and many other androgens are being investigated as potential therapies for functional limitations associated with aging and illness. However, randomized trials have failed to show consistent improvements in functional performance; the effects of testosterone on physical activity also remain poorly understood [7], [8]. In fact, in a recent trial in older men with mobility limitation, testosterone administration failed to induce significant improvements in physical activity [8].

Functional performance is an integrated measure of physical function that has been associated with important health outcomes, including frailty, functional limitations, and mortality. Although there are several potential reasons why testosterone alone may not induce consistent improvements in functional performance in spite of substantial gains in muscle mass, one plausible hypothesis is that physical exercise is required to induce the adaptations necessary for translation of muscle mass gains into functional improvements. Low-intensity physical exercise interventions that emphasize walking improve some aspects of physical performance but have been typically associated with only modest gains in muscle mass and physical function. However, the effects of testosterone administered in conjunction with an adjunctive low-intensity physical exercise on functional performance and bioenergetics are unknown and were the subject of this investigation. In this study, we tested the hypothesis that the combined administration of testosterone plus a low-intensity physical training program in male mice at a late stage of life would improve functional performance and bioenergetics more than low-intensity physical training alone.

Functional performance is an integrated measure of complex interplay of multiple factors, including muscle mass and quality, bioenergetics, behavioral, and social factors. In skeletal muscle, mitochondrial oxidative phosphorylation (OXPHOS) serve as a major source of energy to meet basic metabolic needs and energy demands during physical activity. Recent studies have linked the progressive decline in skeletal mitochondrial function with aging and functional decline, where physical strength declines out of proportion to the loss of muscle mass [9], [10]. Mitochondria are also the major source of free radicals, generated as byproducts from OXPHOS, whose effects are counteracted by multiple scavenging enzymes and non-enzymatic antioxidants. To optimize energy production and minimize oxidative damage, mitochondria are engaged in dynamic network exchange through fission and fusion, which identifies damaged mitochondrial and tags them for mitophagy [11], [12]. Impairments of these mechanisms of mitochondrial quality control during aging contribute to the age-related increase in tissue oxidative damage and functional decline [13], [14], [15], [16], [17]. Aerobic exercise is an effective physiological intervention that counteracts aging-related mitochondrial dysfunction through simultaneous improvement of mitochondrial biogenesis and quality control, including up-regulation of mitophagy [18], [19], [20]. Glass and colleagues reported that testosterone enhances voluntary wheel running in castrated young adult mice and increases the mRNA expression of mitochondrial genes, especially those related to complex I in the electron transport chain [21]. In addition, myocyte-specific transgenic expression of androgen receptor in young adult rats and mice also increases skeletal mitochondrial enzyme activities and increases *in vivo* oxygen consumption [22], [23]; implying a positive role of testosterone and its nuclear receptor on mitochondrial biogenesis and function. Hence a second aim of this study was to determine the effects of testosterone supplementation plus low-intensity physical exercise on muscle mitochondrial biogenesis and quality control. We hypothesized that testosterone supplementation plus low intensity physical exercise training will improve skeletal muscle mitochondrial biogenesis and mitochondrial quality control in very old mice undergoing routine low-intensity physical training.

We used 28-month old male C57BL6 mice as our model because these animals display age-related decline in testosterone levels similar to that observed in older men. The intervention was started in late life - at 28 months of age - and lasted for two months, giving an age range within the 50–25% survival window (http://www.nia.nih.gov/aged-rodent-colonies-handbook/strain-survival-information). Previous studies suggest that functional performance within this survival window predicts late life healthspan in both human and rodents [10], [24]. We show here that testosterone plus low-intensity physical training even at this late stage of life increases spontaneous physical activity, respiration, and grip strength more than physical training alone, in addition to the expected gains in muscle mass. We also provide the first evidence that testosterone supplementation when administered together with low-intensity physical training increases mitochondrial biogenesis and improves mitochondrial quality control.

## Materials and Methods

### Animals

The animal use protocol for this study was approved by the Institutional Animal Care and Use Committee of Boston University School of Medicine. Male C57BL/6 mice at 28 months of age were obtained from the rodent longevity colony of the National Institute on Aging (NIA). After acclimation, baseline measurements of body composition using nuclear magnetic resonance (NMR), grip strength, and treadmill performance (6 m/min, 5% incline) were obtained.

### Testosterone Administration

After completion of baseline assessments, the mice were assigned with matching body composition and grip strength to receive either testosterone or vehicle injection (N = 8 in each group). Testosterone, dissolved in medium-chain oil (www.life-enhancement.com), was administered by subcutaneous injection (50 mg/kg, twice per week). Control mice were injected with equal volume of oil (100 ul). Serum testosterone concentration was measured using liquid chromatography tandem mass spectrometry (10–20 ng/dL for control mice and 500- 1000 ng/dL for mice injected with testosterone).

### Low-intensity Physical Training Routines

Both control and testosterone-treated animals were engaged in a 30 min treadmill walk three times each week (6 m/min, 5% inclination) for the first six weeks. This condition is considered as “low-intensity” as compared to a 60 min daily running at 13 m/min, 10% incline for similarly aged rodents [18]. To promote walking, a shock grid (0.97 mA, 3 Hz) at the back of the treadmill was used to discourage the mice from stopping while the treadmill belt was moving. After the initial acclimation, all mice walked voluntarily throughout 30 min without the use of the shock grid. Two mice in the control group died before and one died after the final functional assessments. Hence, for most of the functional tests, we had 6 to 8 mice in each group but tissues were available for 5 mice in the control group and 8 mice in the testosterone group. At the end of the experiment, all animals were examined for abnormality for internal organs and external appearance. Most animals show moderate discoloring in seminal vesicles with no difference between the control and testosterone supplemented groups. No incidence of visually detectable tumor, ulcer, or other abnormal tissue appearance was found among these animals.

### Functional Assessment

NMR and grip strength measurement was repeated in week 7. Metabolic cage study was performed at the end of week 6 and rotarod test during week 7, as described previously [25]. For grip strength, the animals were allowed to grasp a horizontal metal bar while being pulled by their tail. The results were recorded using an automatic force transducer (Columbia Instrument, www.colinst.com). For rota-rod test, a 4-lane accelerating rota-rod (Columbus Instrument), equipped with a built-in automatic timer was used and the speed of the rotating rod was adjusted manually. Mice were trained first at a low speed starting at 4 rpm, and the speed was gradually increased by 0.5 rpm/min to 9 rpm over 3 days. For the running test, mice were placed on a static rod. Once all animals were on the rod, the motor was turned on and the rod rotation was accelerated at a rate of 0.5 rpm/min until all animals fell off the rod. The running distance was calculated as the speed multiplied by the running time.

### Tissue Analysis

Tissue DNA and RNA isolation, RNA reverse transcription, and real-time qPCR were performed as described before [25]. All PCR primers were designed as intron-spanning except for the nuclear DNA-encoded cytochrome c and all mitochondrial DNA-encoded genes which do not have introns. To eliminate DNA contamination for qPCR analysis of these genes, mRNA was isolated from total RNA samples using the mRNA purification kit (Qiagen #72041). We found this method to be superior to the DNase I digestion method which was efficient in eliminating nuclear DNA but not mitochondrial DNA. The mRNA thus obtained was reverse-transcribed to first-strand cDNA using standard protocol. For Western analysis, tissue was homogenized in cell lysis buffer (Cell Signaling Technology, #9803, www.cellsignal.com) supplemented with 0.1% SDS and standard cocktails of protease inhibitors and phosphatase inhibitors and 10 mM DTT. Lysate was cleared by centrifuge at 3000 g for 15 min. Protein was loaded as 0.015 mg/lane for detection of mitochondrial proteins and 0.05 mg/lane for detection of other proteins. A special blocking reagent (Rodent block M, www.biocare.net) was used to block endogenous mouse IgG signals. A light-chain specific anti-mouse secondary antibody (Millipore, #MAB201P) was used for all first antibodies that were generated in mice. Mitochondria-enriched fraction was isolated from frozen skeletal muscle following a published protocol [26]. The protocol was validated in-house using Western analysis to confirm a substantial enrichment of the mitochondrial enzyme manganese superoxide dismutase (MnSOD) and a marked reduction in alpha-actin concentration in the mitochondrial fraction as compared to a whole tissue lysate. Mitochondrial protein ATP synthase subunit 5a (ATP5a) was used as loading control for mitochondria-enrich fractions and GAPDH was used for loading control for whole tissue lysates. Antibodies for mitoprofile cocktail were purchased from Mitoscience (www.mitoscience.com, #MS604) and antibody for GAPDH and secondary antibody against mouse or goat IgG from Santa Cruz (www.scbt.com). All other first antibodies and a secondary against rabbit were from Cell Signaling Technology. Results of Western analysis were quantified using NIH Image J program. Thiobarbituric Acid-Reactive (TBAR) substance was measured in skeletal muscle lysate using a commercial kit following manufacturer’s instructions (www.caymanchem.com, # 10009055). To avoid tissue oxidation during sample preparation, butylated hydroxyanisole was added to a lysis buffer to a final concentration of 5 mM.

### Assays for Mitochondrial Enzyme Activity

Frozen tissue was pulverized in liquid nitrogen. About 30 mg of tissue was homogenized in 2 ml buffer (20 mM Tris, sucrose 250 mM, KCl 40 mM, EGTA 2 mM, 10% glycerol (v/v) and 1 mM PMSF). A Glas-Col motor-driven homogenizer was used at 4000 rpm and each sample was homogenized by 30 strokes in a standard homogenizer with fitted Teflon pestle, with homogenizer kept on ice all time. The solution was then separated into 2×0.8 ml fractions. For samples to be used for citrate synthase assay, 0.04% Triton X-100 was added. For samples to be used for mitochondrial electron transport chain activity, 0.02% dodecyl maltoside was added. Both fractions were rotated at 4°C for one hour and then centrifuged at 3000 g for 15 min. The supernatant was divided into small aliquots and stored at −80°C until use. The enzyme activities were measured using standard spectrophotometry.

### Statistical Analyses

Results are presented as mean ± SEM. Comparison between two groups were analyzed using Student’s t test. Comparisons among multiple groups were performed using one-way analysis of variance (ANOVA), followed by Tukey’s test.

## Results

### 1. Testosterone Supplementation Plus Low-intensity Physical Training Improves Functional Performance in Aging Male Mice

**Figure 1:**
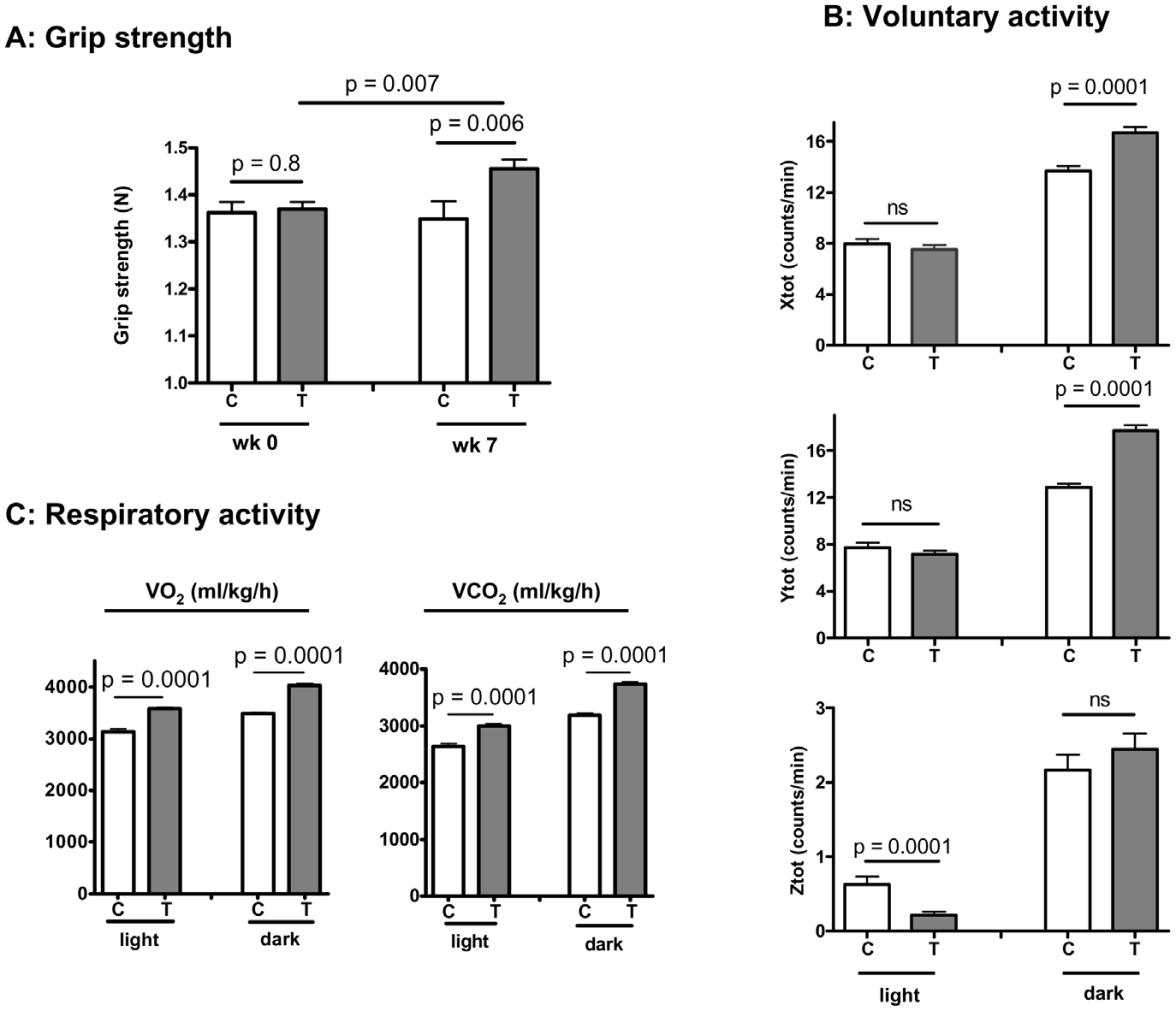
Effect of testosterone supplementation plus low-intensity physical training on grip strength, spontaneous physical activity, and respiratory activity in old male mice. (**A**) Forelimb grip strength measured at baseline (wk 0, N = 8 per group, C: control, T: testosterone) and after 7 weeks of (N = 6 for control, N = 8 for T group) testosterone supplementation (10 mg/kg, sc, twice per week). Control was injected with an equal volume of vehicle (0.1 ml). (**B**) Spontaneous physical movements recorded as total counts of infrared beam breaking per min. Each animal was housed individually in a metabolic cage. Results were averaged separately over the light and dark periods. The measurement was performed during week 6 after testosterone supplementation (N = 4 for each group). Xtot, Ytot, Ztot each denotes movements in X-axis, Y-axis, and Z-axis. (**C**) Mean respiratory activity recorded as oxygen consumption and carbon dioxide production for animals individually housed in a metabolic cage (N = 4). Results were normalized to total body weight. All results are shown as mean ± SEM, and the difference between control and testosterone groups at each time period was analyzed by *t* test. ns, not significantly different.

As expected,, testosterone plus low-intensity physical training (**T/PT**) for two months beginning at 28 month of age was associated with a significant increase in whole body lean mass and a decrease in fat mass, in comparison to the baseline values or by cross-comparison with the control group that received vehicle and the same level of physical training (**V/PT**). These results are shown in supplementary Figure S1A. The mice receiving **T/PT** also had significantly higher mass of weight-bearing forelimb (triceps) and hind-limb (quadriceps femoris and gastocnemius/soleus) muscles, as well as non-weight-bearing cardiac, pectoralis, and levator ani muscles than those receiving **V/PT** (Figure S1B). The mice receiving **T/PT** displayed a significant increase in grip strength over the 7 week treatment period, while the control mice showed no change (Figure 1A, left panel). The mice receiving **T/PT** also displayed an average 30% increase in Rota-rod running distance, although the effect did not reach statistical significance (Figure S2). When placed in a metabolic cage system, **T/PT** mice displayed a significant increase in their nocturnal spontaneous movements along the X- and Y- axes (Figure 1B, upper and middle panels) but not in the Z-axis (Figure 1B, lower panel). Diurnal activity was not significantly different between the two groups in the X- and Y-direction but was lower in the Z-axis for the **T/PT** group. Since mice are nocturnal creatures, this difference in the day time may not be physiologically important.

In comparison with the **V/PT** group, treatment with **T/PT** increased O_2_ consumption and CO_2_ production by 14.3% and 13.5% during day time and by 17.9% and 20.9% at night time, respectively, after normalization by body weight (Figure 1C). As O_2_ consumption is related to lean mass and mitochondrial function, and each may contribute to increase respiration independently [27], we also normalized the data by lean body mass and found that the increase in respiratory activity in **T/PT** group remained significant (Figure S3). The increase in respiration induced by **T/PT** was observed both during the dark period and the light period when the animals were less active (Figure S4); indicating that part of the increase in metabolic rate was independent of muscle mass and physical movements. The respiratory exchange ratio was similar between the two groups and varied from near 0.8 in the light period to near 0.95 in the dark period (data not shown). Together, these results indicate that late-life testosterone supplementation plus low-intensity physical training improved functional performance as compared to the control animals that received the vehicle but engaged in the same physical training protocol.

### 2. Testosterone Supplementation Plus Low-intensity Physical Training Increases Mitochondrial DNA Copy Number and the Expression of Selected Mitochondrial Transcripts in Skeletal Muscle of Aging Male Mice

**Figure 2:**
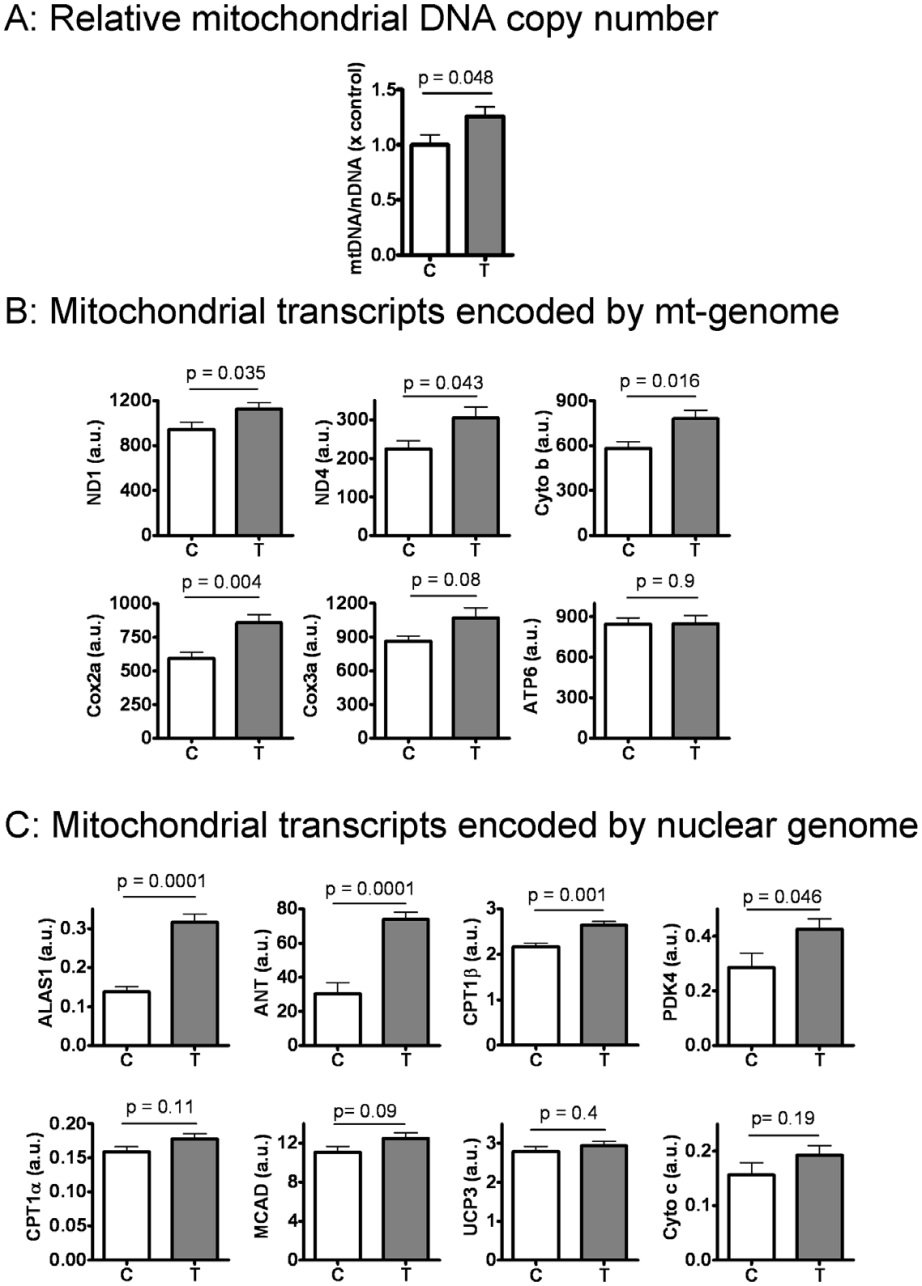
Effect of testosterone supplementation plus low-intensity physical training on markers of mitochondrial biogenesis in the skeletal muscle. (**A**) Testosterone increased mitochondrial DNA (mtDNA) copy number when normalized to nuclear genome DNA (nDNA). (**B**) Testosterone increased mtDNA-encoded mitochondrial transcripts for markers of complex I (NADH dehydrogenase subunits: ND1, ND4), complex III (cytochrome b: Cyto b), complex IV (cytochrome c oxidase subunits: Cox2a and Cox3a), and complex V (ATP synthase subunit 6, APT6) of the electron transport chain. (**C**) Testosterone increased the expression of selected nuclear genome-encoded mitochondrial transcripts for δ-aminolevulinate synthase 1 (ALAS1), adenine nucleotide translocator (ANT), Carnitine palmitoyltransferase 1(CPT1β, CPT1α). pyruvate dehydrogenase kinase 4 (PDK4), medium-chain acylCoA dehydrogenase (MCAD), uncoupling protein 3 (UCP3), and cytochrom c (Cyto c). All qPCR results were normalized to house-keeping gene Hypoxanthine phosphoribosyltransferase (HPRT) and expressed in an arbitrary unit (a.u). All results were obtained from triceps and presented as mean ± SEM and compared by *t* test. N = 5 for the control (C) and N = 8 for the testosterone (T) group.

Skeletal muscle force capacity is correlated with mitochondrial content and function [28]. Administration of **T/PT** was associated with a significantly greater (20% higher) mitochondrial DNA copy number per unit nuclear DNA (mtDNA/nDNA ratio) than **V/PT**. We measured the mRNA expression of selected mitochondrial transcripts encoded by the mitochondrial as well as the nuclear genomes. As shown in Figure 2B, **T** **/PT** increased the expression of genes encoded by mtDNA, including NADH dehydrogenase subunits (complex I: ND1, ND4), cytochrome b (complex III), and cytocrhome c oxidase subunits (complex IV: Cox2a and Cox3a), but not ATP synthase subunit 6 (ATP6, complex V). Among the tested mitochondrial transcripts encoded by nDNA, **T/PT** induced a large increase in aminolevulinate, δ-synthase 1, (ALAS1, mitochondrial heme synthesis [29]) and adenine nucleotide translocase (ANT, exchanges cytosolic ADP for mitochondrial ATP). **T/PT** also moderately increased the mRNA expression of CPT1beta (gate-keeper for mitochondrial fatty acid oxidation) and PDK4 (inhibitor of pyruvate oxidation to favor fatty acid for mitochondrial fuel [30]). Neither the expression of cytochrome c, a nuclear genome encoded component of complex IV, nor that of UCP3, a muscle-specific mitochondrial uncoupling protein, was significantly affected. The latter finding agrees with others showing that testosterone does not increase UCP3 expression in myotubes from male donors [31]. Together, these results show that testosterone supplementation increased the mtDNA copy number and also the expression of selected mitochondrial transcripts encoded by both mitochondrial and nuclear genomes in the old mice that were engaged in low-intensity physical training.

### 3. Effects of Testosterone Plus Low-intensity Physical Training on the Expression of Selected Mitochondrial Proteins and the Activity of Selected Enzymes in Skeletal Muscle of Aging Male Mice

**Figure 3:**
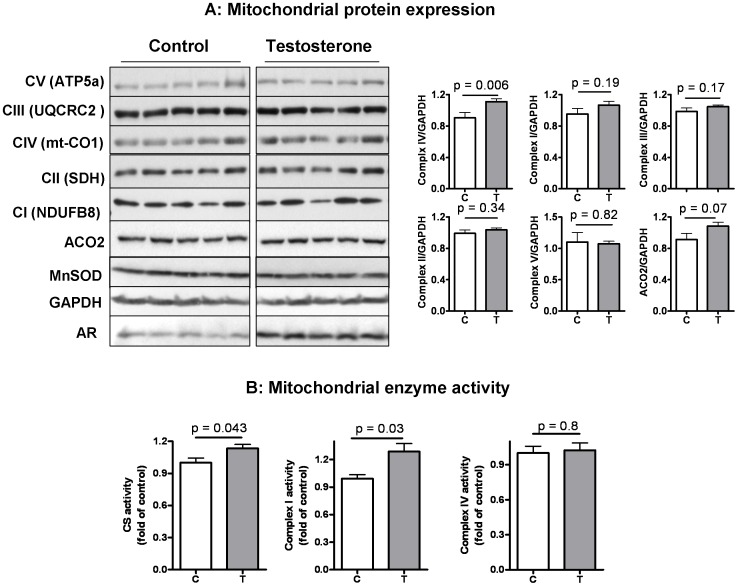
Effect of testosterone supplementation plus low-intensity physical training on the expression of mitochondrial proteins and the activity of mitochondrial marker enzymes in skeletal muscle. (**A**) (left panel) Western analysis of a panel of subunits of complex I-V as well as mitochondrial aconitase (ACO2) and superoxide dismutase (MnSOD). GAPDH was used as loading control and expression of androgen receptor was measured as a positive control; (right panel) quantification of protein expression normalized to GAPDH. (**B**). Activity of mitochondrial enzyme citrate synthase, complex I, and complex IV. All measurements were performed using whole tissue homogenates of the triceps muscle group. Bar graphs are presented as mean ± SEM and group means were compared using Student’s *t* test, N = 5 for the control (C) group and 8 for the testosterone (T) group.

We measured the protein expression levels of selected subunits of each complex in the mitochondrial electron transport chain (ETC). When normalized to the loading control GAPDH, **T/PT** was associated with an increase in the expression of complex IV subunit (mt-CO1) (Figure 3A). However, the expression levels of selected subunits of complex I (NDUFB8), complex III (UQCRC2), complex II (SDH), and complex V (ATP5a) did not differ significantly between the control and testosterone groups. The increase in mt-CO1, encoded by the mtDNA, induced by **T/PT** is consistent with increased transcripts of Cox2a and Cox3a, two other complex IV subunits also encoded by mtDNA (Figure 2). We also measured the expression level of selected non-ETC mitochondrial proteins, such as aconitase (ACO2) and superoxide dismutase (MnSOD); both were not significantly affected by **T/PT** (Figure 3A). As a positive control, skeletal muscle of the **T/PT** group was found to associate with a higher androgen receptor (AR) expression level than that of the **V/PT** group, as expected (Figure 3A).

As shown in Figure 3B, **T** **/PT** increased the activity of citrate synthase and complex I, but did not affect complex IV. Citrate synthase is the flux-generating enzyme in the citric acid cycle and complex I is the primary site of electron entrance to the mitochondrial ETC, whereas complex IV is the last site before energy is released to drive ATP synthesis. The lack of an effect on complex IV was somewhat unexpected, considering that complex IV transcripts and protein markers (cox 2a, cox 3a, and mt-CO1, all encoded by mtDNA) were increased by **T/PT**. In contrast, complex I displayed increased activity but lack of change in protein expression for its subunit marker (NDUFB8, encoded by nDNA), although the expression of mtDNA-encoded subunits of complex I (e.g. ND1 and ND4) was increased by **T/PT** (Figure 2). Because each complex contains multiple subunits encoded by both genomes, and we only measured one subunit for each complex, it is possible that the selected markers may not be rate-limiting factor for the specific enzyme complex. A recent microarray analysis showed that wheel running plus testosterone administration in mice after orchidectomy up-regulated a large number of nuclear genome-encoded transcripts for complex I, but only one for complex IV [21], implying that testosterone plus physical activity intervention may have a greater impact on complex I than complex IV. This is in line with our data on the enzyme activity measurements displayed in Figure 3B.

### 4. The Effects of Testosterone Plus Low-intensity Physical Training on the Expression of Biomarkers of Mitochondrial Fusion and Fission as well as Key Elements in Mitophagy in Skeletal Muscle of Aging Male Mice

Thus far, our data show that **T/PT** improved physical activity and metabolic rate (Figure 1A–C), and was associated with higher mtDNA copy number, increased expression of selected mitochondrial transcripts (Figure 2A–C), and increased activity of citrate synthase and complex I (Figure 3B). However, these changes did not correlate robustly with changes in mitochondrial protein expression levels. We hypothesized that the increase in mitochondrial biogenesis might have been balanced by a parallel increase in dynamic mitochondrial turnover, e.g. mitophagy [32]. Aging is known to be associated with a general decline in autophagy and a specific decline in mitophagy in skeletal muscle [14], [33], [34], [35], whereas intensive endurance exercise can increase skeletal muscle expression of markers for mitophagy and offset age-related decline in muscle mitochondrial quality control [18], [36]. We considered the possibility that **T/PT** increases mitochondrial quality control, namely mitochondrial fussion, fission, and mitophagy, in addition to stimulating mitochondrial biogenesis, which could explain the observed functional improvement in the absence of a significant increase in mitochondrial protein mass.

**Figure 4:**
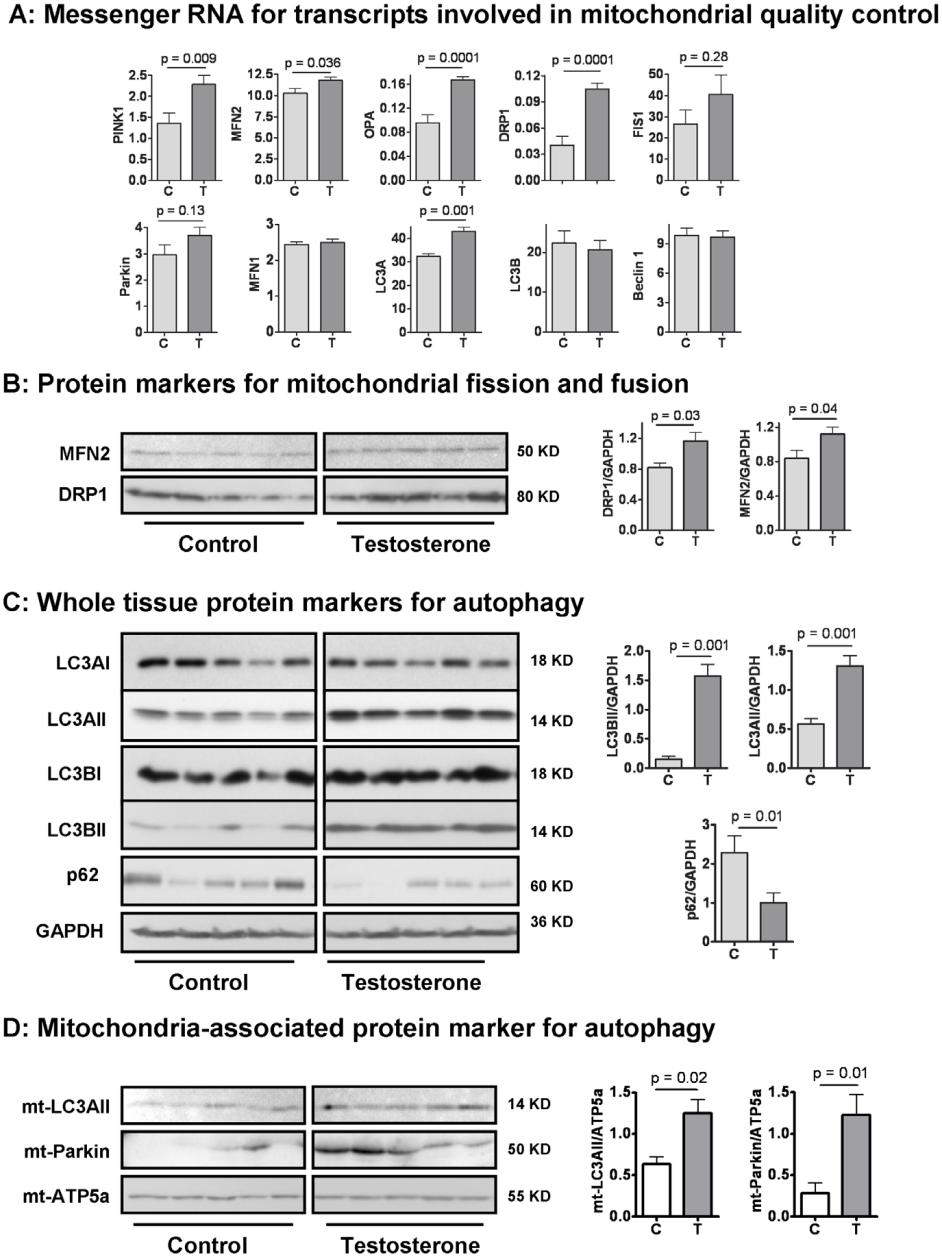
Effect of testosterone supplementation plus low-intensity physical training on mitochondrial quality control in skeletal muscle. (**A**) Testosterone increased the mRNA expression of selected transcripts involved in mitochondrial fission, fusion, and mitophagy. (**B**) Testosterone increased the expression of mitochondrial fusion protein mitofusin 2 (MFN2) and mitochondrial fission protein DRP1; both are normalized to GAPDH. (**C**) Testosterone increased protein expression of autophagasome marker LC3II in whole tissue homogenates with a reciprocal decrease in the protein expression of autophagasome adaptor/substrate SQSMT1/p62. (**D**) Testosterone increased the expression of LC3II and Parkin in the mitochondria-enriched fraction of skeletal muscle lysate. Each lane represents one individual animal. The data in the bar graphs are mean ± SEM of desitometry results, and analyzed by *t* test. N = 5 for the control (C) group and 8 for the testosterone (T) group (for visual clarity, only five are displayed in Western blots).

To test this hypothesis, we measured the expression of several transcripts involved in mitochondrial quality control, including the key elements in mitochondrial fission, fusion, and mitophagy. Damaged mitochondria are targeted for mitophagy after being removed from the dynamic fission and fusion cycles. Fusion is regulated primarily by mitofusin 1 and 2 (MFN1, MFN2) and OPA1, and fission by DRP1 and Fis1 [37], [38], [39], [40]. As shown in Figure 4A, **T** **/PT** increased mRNA expression of MFN2, OPA1, DPR1, and Fis1. Western analysis also revealed an increase in protein expression of MFN2 and DRP1 (Figure 4B). These results support the hypothesis that **T/PT** increases mitochondrial fission and fusion, which would be expected to enhance mitochondrial proliferation and as well as to eliminate damaged organelles.

Dysfunctional mitochondria are tagged by PINK1, which recruits Parkin for self-ubiquitination to be targeted for autophagasome, which eventually fuses with the lysosome for hydrolytic degradation [41], [42]. As shown in Figure 4A, **T** **/PT** increased mRNA expression of PINK1 but did not affect Parkin. However, Parkin, a component of the E3 ubiquitin ligase, is generally regulated at a post-translational level. To test the hypothesis that **T/PT** may promote mitophagy, we measured the protein expression of the autophagasome marker LC3II in skeletal muscle whole tissue lysate and mitochondria-enriched fractions. **T/PT** was associated with increased expression of LC3AII and LC3BII in the whole tissue (Figure 4C) and in the mitochondria-enriched fractions (Figure 4D), indicating an increase in total and mitochondria-associated autophagosomes [43], [44], [45], [46]. Parkin, which ubiquitinates mitochondrial to initiate autophagasome formation [41], was also increased in the mitochondria-enriched fraction (Figure 4D). These data suggest that **T/PT** increased the formation of mitochondria-associated autophagasomes. We cannot determine from these data whether autophagic flux was increased or decreases, as both events could result in a quantitative increase of autphagasomes in a steady-state. However, if the flux were decreased, one would expect to detect a non-specific increase in other autophagic proteins. However, Western analyses did not reveal significant changes in beclin 1, Atg7, and Parkin expression levels in whole tissue lysate (data not shown), pointing against a general blockade of autophagic flux in response to **T/PT**. Finally, we show that the expression of autophagic receptor, SQSTM1/p62, was markedly reduced in response to **T/PT** (Figure 4C). As one of the “classical” autophagic receptor, p62 is known to mediate “selective” autophagy, including mitophagy [47]. Being located in the lumen of autophagasomes, p62 itself is an autophagy substrate which is degraded together with the cargo it carries [48]. Hence, a reduction of p62, coupled with an increase in upstream autophagasome number (marked by LC3II), suggest that **T/PT** increased the formation of mitochondrion-associated autophagasomes, and enhanced their removal through increased autophagic flux [48], [49], [50].

### 5. Testosterone Plus Low-intensity Physical Training Reduces Tissue Oxidative Damage, Attenuates Stress Kinase Activation, and Increases Pro-myogenic and Anti-inflammatory Growth Factors in Skeletal Muscle of Aging Male Mice

**Figure 5:**
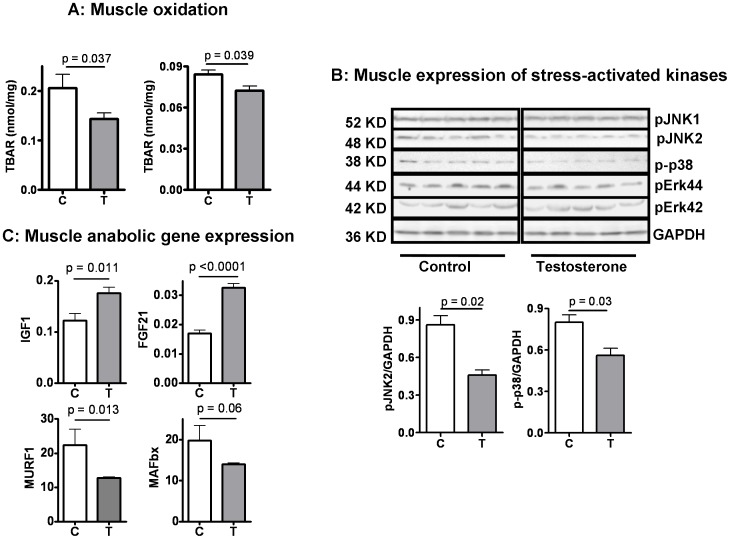
Effect of testosterone supplementation plus low-intensity physical training on protein oxidation/carbonylation and phosphorylation/activation of stress-activated kinases in skeletal muscle. (**A**) Testosterone reduced skeletal muscle thiobarbituric acid reactive substance (TBAR) in quadriceps (left) and gastrocnemius (right) muscle homogenates, implying a reduction in tissue oxidant stress. (**B**) Testosterone reduced phosphorylation of the two stress-activated protein kinase JNK2 and p38, without affecting the mitogen-activated Erk42/44; serving as a second line of evidence for a reduction in tissue stress in response to testosterone supplementation plus low-intensity physical training interventions. (**C**) Testosterone increased mRNA expression of IGF1 and FGF21 while reduced mRNA expression of MURF1 and MAFbx in skeletal muscle (triceps). The data in the bar graphs are mean ± SEM and analyzed by *t* test. N = 5 for the control (C) group and 8 for the testosterone (T) group (for visual clarity, only five are displayed for each group in the Western blots).

Dysfunctional mitochondria are the primary source of free radicals that contribute to the age-related oxidative damage in skeletal muscle [51]. If **T/PT** improves mitochondrial biogenesis and quality control as our data suggest, the tissue oxidant stress would be expected to be reduced as well. To test this hypothesis, we measured the effect of **T/PT** on tissue content of thiobarbituric acid reactive substances (TBAR). Since the TBAR assay measures the products of lipid peroxidation, it is particularly useful for assessing cumulative oxidative damage in the tissue and the interpretation is less confounded by intervention-related changes in protein degradation pathways including proteasomes and autophagasomes. As shown in Figure 5A, TBAR was reduced in both quadriceps and gastrocnemius muscle groups of animals receiving **T/PT** in comparison to the control group that received **V/PT**. This observation was associated with a reduction in phosphorylation (activation) of stress-activated protein kinase JNK2 and p38-Map kinase in skeletal muscle from mice treated with **T/PT** (Figure 5B)**.** A third member of MAPK family, Erk42/44, was not different between **V/PT** and **T/PT** groups (Figure 5B). While all three members of the MAPK family can be activated by acute peroxide induction, phosphorylation/activation of JNK and p38 are more sustained in the presence of long-term stress while Erk42/44 activation is usually transient and more responsive to growth factor stimulation [52], [53], [54], [55]. Together, these results suggest that treatment with **T/PT** reduced tissue oxidative stress in skeletal muscle. Concurrently, skeletal muscle from **T/PT** group also showed increased expression of insulin-like growth factor IGF1 and reduced expression of the muscle atrogenes, MuRF1 and MAFbx, as shown in Figure 5C. **T/PT** was also associated with increased mRNA expression of fibroblast growth factor 21 (FGF21, Figure 5C), an anti-inflammatory myokine that has been shown to correlate with serum testosterone concentrations [56]. Both IGF1 and FGF21 are known to be negatively regulated by oxidant stress in muscle cells [57], [58]. As both IGF1 and FGF21 are closely related to optimal muscle function and systemic metabolism [59], [60], increased expression of these growth factors can be another useful marker for improved muscle quality in response to **T/PT** treatment. It remains to be determined whether there is a cause-and-effect link between these growth factors and **T/PT-**mediated improvement in skeletal muscle mitochondrial biogenesis and quality control.

## Discussion

Testosterone supplementation administered late in life, in the setting of low-intensity physical training, improved spontaneous physical activity, metabolic rate, and grip strength. Importantly, we show that testosterone supplementation to very old mice plus low-intensity physical training increased mitochondrial biogenesis in the skeletal muscle, with concurrent increase in the expression of markers of mitochondrial fission, fusion, and mitophagy, resulting in a significant reduction in markers of tissue oxidant stress. These data collectively suggest that testosterone plus low-intensity physical training increased mitochondrial biogenesis and accelerated the removal of damaged mitochondria, resulting in better skeletal muscle quality, which could contribute to the improvement of functional performance, reflected in the improvement of physical function. Previous studies have shown that functional performance in a compressed time window of late life predicts healthspan and longevity [10], [24]. Since increasing healthspan is the ultimate goal of anti-aging interventions, future studies should determine the effects of testosterone plus low-intensity physical training on healthspan in older human adults.

Because all animals involved in this study had received low-intensity physical training, we cannot determine from the current data whether testosterone administration alone would produce similar improvements in physical activity and mitochondrial function. In a preliminary study in which 28-month old mice were given testosterone supplementation for 3 weeks without exercise training, we found no effect of testosterone supplementation on mitochondrial DNA copy numbers although an increase in muscle mass was significant. Unfortunately, no functional performance was measured in these animals. Randomized trials of testosterone in older men with mobility limitation have not found significant changes in physical activity [8]. Hence, we suggest that testosterone supplementation alone can increase muscle hypertrophy but may not be sufficient to improve muscle bioenergetics and functional performance in the absence of physical training. Randomized trials in humans are needed to determine the interactive effects of testosterone and low-intensity exercise on functional performance and bioenergetics, to determine the optimal intensity and frequency of physical training that will induce clinically meaningful functional improvements.

Although testosterone has been used for several decades by athletes and recreational body builders to enhance muscle mass and performance, and is being explored as a function promoting therapy for functional limitations associated with aging and chronic illness, the effects of testosterone on mitochondrial biogenesis and function have not been well characterized. Testosterone has been reported to promote, inhibit, or have no effect on mitochondrial biogenesis or function [61], [62], [63], [64], [65]. In men, however, low testosterone levels have been associated with decreased expression of mitochondrial genes in OXPHOS pathway and reduced maximal aerobic capacity [66]. Orchidectomy in young male mice down-regulated gene expression in the pathways for energy metabolism, especially those involved in OXPHOS and ubiquinone pathways [21]. These reports are in-line with our current findings that testosterone supplementation plus low-intensity physical training increased skeletal muscle mitochondrial biogenesis in aging skeletal muscle.

We have provided several lines of evidence that increased mitochondrial biogenesis in response to testosterone plus low-intensity physical training is accompanied by enhanced autophagic removal of damaged organelles. First, testosterone plus physical training increased expression of Parkin and LC3II in the mitochondria-enriched fraction of skeletal muscle lysate, implying increased formation of mitochondria-associated autophagasome. Second, testosterone plus physical training reduced the level of autophagasome adaptor STSTM1/p62 in the muscle, which coupled with an increase in LC3II, the upstream quantitative marker for autophagasome formation, suggests an enhancement of autophagic flux [50]. Third, the activity of selected mitochondrial enzymes was increased by testosterone plus physical training in the absence of a substantial increase in steady-state mitochondrial protein level, suggesting possible improvement in the quality of mitochondrial proteins. Fourth, a reduction in tissue oxidative damage and decreased expression of stress kinases, as well as increased expression of pro-myogenic and anti-inflammatory growth factors and reciprocal reduction in muscle expression of atrogenes also indicate that testosterone plus low-intensity physical training improved muscle quality, which is in-line with an improvement of mitochondrial quality control [13], [32], [67]. Together, our results support the hypothesis that testosterone plus low-intensity physical training improved mitochondrial biogenesis and quality control in old male mice, although the molecular mechanisms underlying these effects remain to be investigated.

In summary, we have provided multiple lines of evidence that links the functional performance with improved skeletal muscle mitochondrial biogenesis and quality control in aging mice treated with testosterone supplementation plus low-intensity exercise as compared with control mice receiving vehicle and low-intensity exercise only. We used older male mice that were naturally testosterone deficient to increase the translational value of the findings to testosterone trials in older men with age-related decline in testosterone levels. Further studies are needed to determine the molecular mechanisms for these observations and to determine the minimal requirements in exercise intensity and frequency that can induce the functional improvements in response to testosterone supplementation.

## Supporting Information

Figure S1 **Effect of testosterone supplementation on body composition. (A).** Body fat mass (upper left), lean mass (upper right), total body weight (lower left) and lean/fat ratio (lower right). Results are shown as means +/− se, N = 8 for control (C28) and testosterone (T28) at baseline. N = 6 for control (C30) and N = 8 for testosterone (T30) group at 30 month, unpaired *t* test. (B). Terminal tissue weight for selected muscle groups as labeled [means +/− se, N = 5 for the control (C) group, N = 8 for testosterone (T) group, unpaired *t* test].
(PPT)

Figure S2 **Effect of testosterone supplementation on rotarod running distance.** Mice were allowed to run on the rota-rod set with a low and gradually increasing speed until they fell off the rod, as described in the Methods. Each data point represented the mean distance by one individual animal. Unpaired *t* test.
(PPT)

Figure S3 **Effect of testosterone supplementation on respiration after normalized to body lean mass.** Results are re-plotted from original data presented in Figure 1C (respiratory activity normalized to total body weight) and Figure S1 (lean mass).
(PPT)

Figure S4 **Comparison of effect of testosterone supplementation on respiration normalized to body weight and lean mass.** Percentage wise, the difference were similar during light period but was diminished during dark period when the results were normalized to lean body mass. Blue: vehicle control; red: testosterone supplementation.
(PPT)
