# Supplementary figures and images for: Testosterone Plus Low-Intensity Physical Training in Late Life Improves Functional Performance, Skeletal Muscle Mitochondrial Biogenesis, and Mitochondrial Quality Control in Male Mice
Source: PLoS One. 2012 Dec 11;7(12):e51180. doi: 10.1371/journal.pone.0051180 (PMC3519841; doi:10.1371/journal.pone.0051180)

## Slide 1
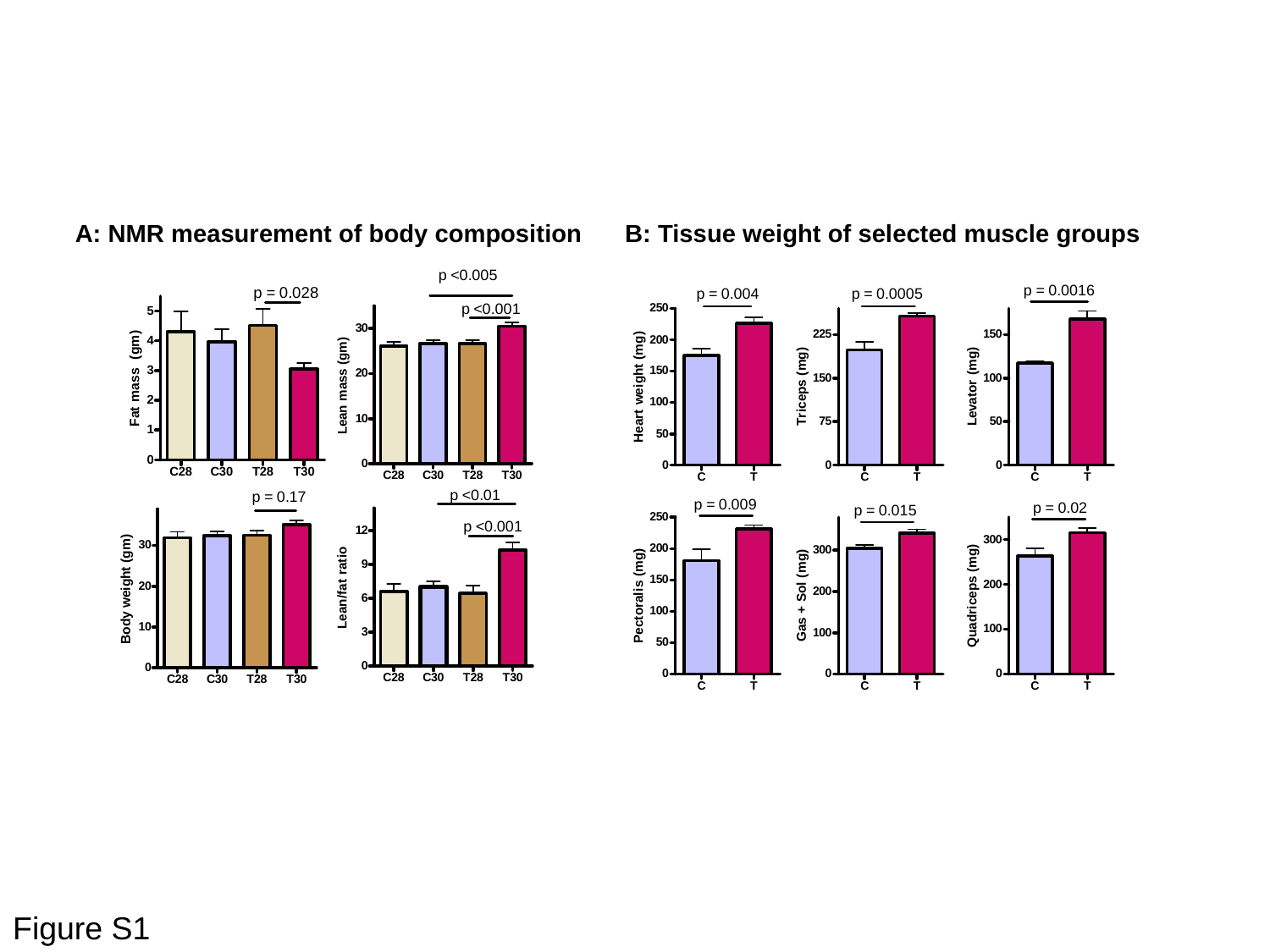

A: NMR measurement of body composition
B: Tissue weight of selected muscle groups
Figure S1

Supplement: Figure S1 — Effect of testosterone supplementation on body composition. (A). Body fat mass (upper left), lean mass (upper right), total body weight (lower left) and lean/fat ratio (lower right). Results are shown as means +/− se, N = 8 for control (C28) and testosterone (T28) at baseline. N = 6 for control (C30) and N = 8 for testosterone (T30) group at 30 month, unpaired t test. (B). Terminal tissue weight for selected muscle groups as labeled [means +/− se, N = 5 for the control (C) group, N = 8 for testosterone (T) group, unpaired t test]. (PPT) [file pone.0051180.s001.ppt]

## Slide 1
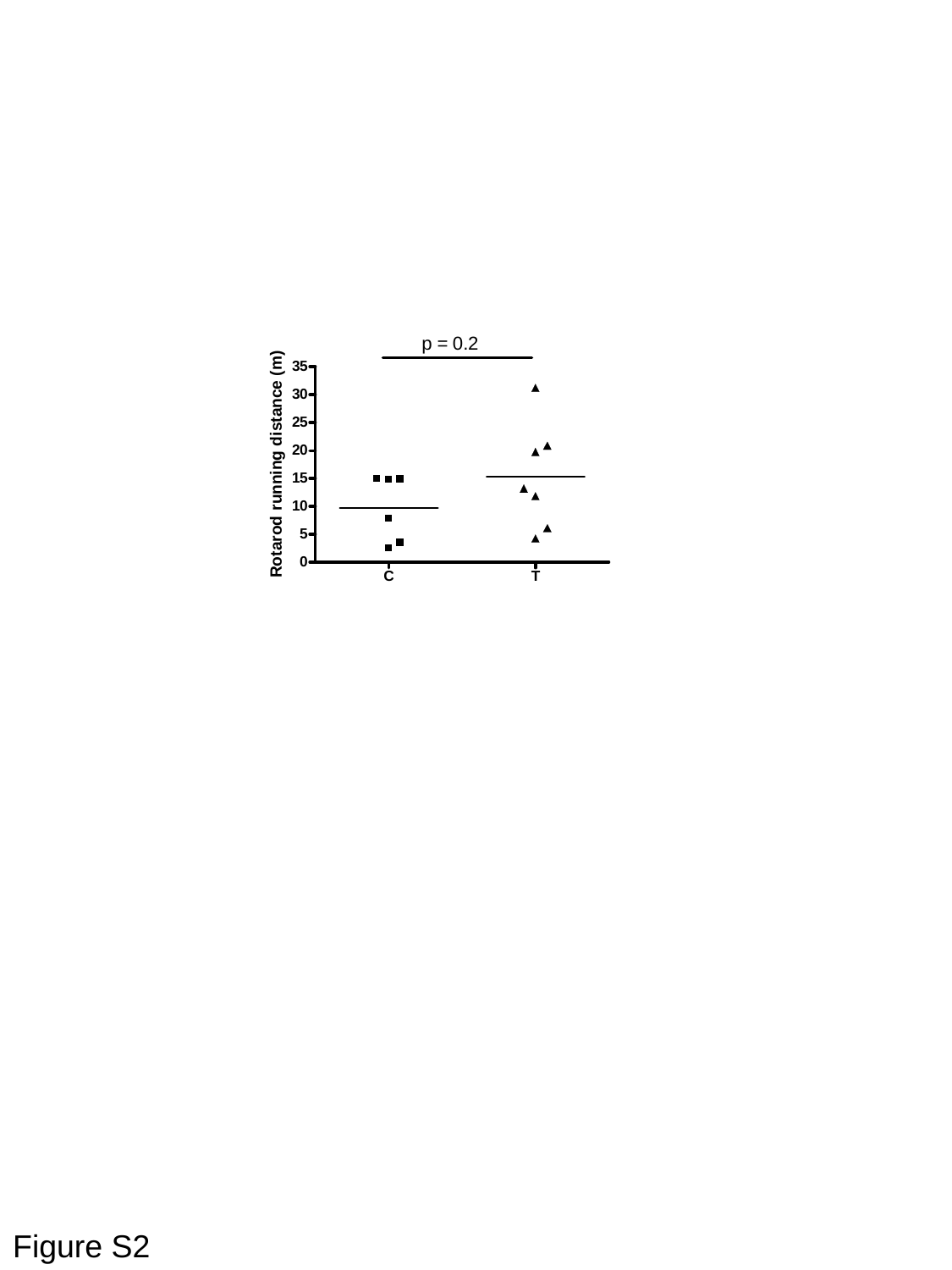

Figure S2

Supplement: Figure S2 — Effect of testosterone supplementation on rotarod running distance. Mice were allowed to run on the rota-rod set with a low and gradually increasing speed until they fell off the rod, as described in the Methods. Each data point represented the mean distance by one individual animal. Unpaired t test. (PPT) [file pone.0051180.s002.ppt]

## Slide 1
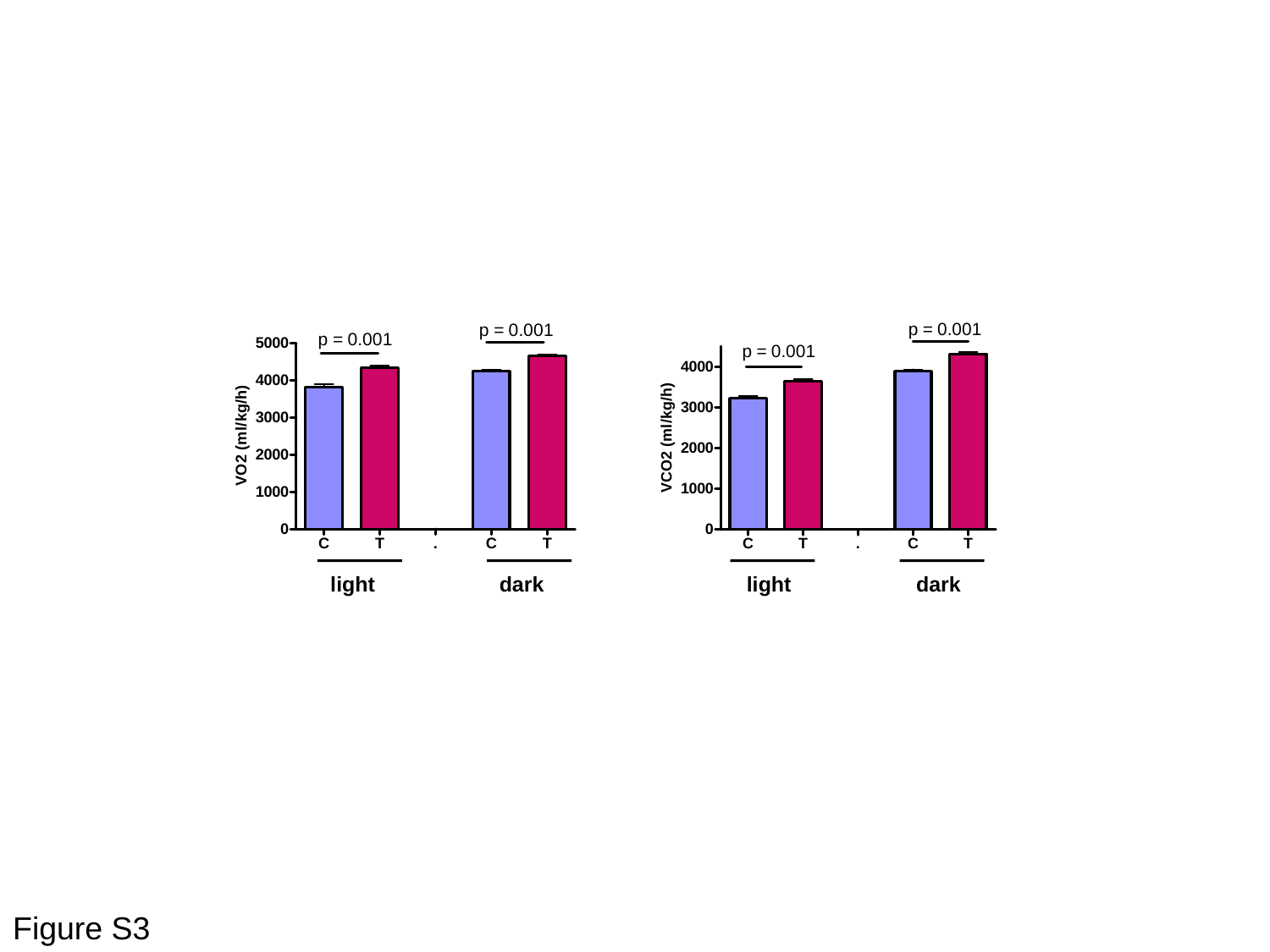

light
dark
light
dark
Figure S3

Supplement: Figure S3 — Effect of testosterone supplementation on respiration after normalized to body lean mass. Results are re-plotted from original data presented in Figure 1C (respiratory activity normalized to total body weight) and Figure S1 (lean mass). (PPT) [file pone.0051180.s003.ppt]

## Slide 1
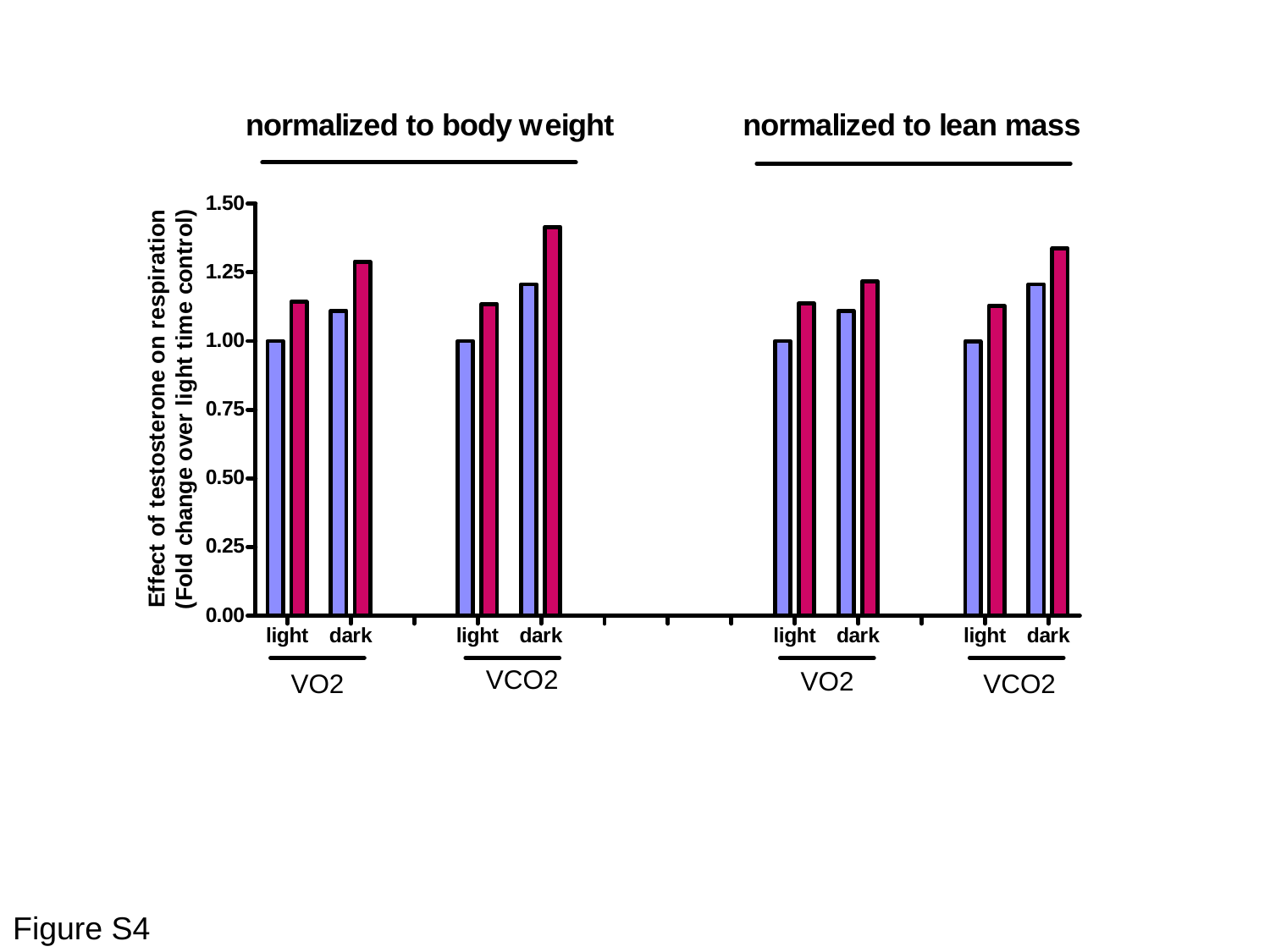

Figure S4

Supplement: Figure S4 — Comparison of effect of testosterone supplementation on respiration normalized to body weight and lean mass. Percentage wise, the difference were similar during light period but was diminished during dark period when the results were normalized to lean body mass. Blue: vehicle control; red: testosterone supplementation. (PPT) [file pone.0051180.s004.ppt]
